# The Association Between Gastroesophageal Reflux Disease and Lung Cancer: A Systematic Review and Meta Analysis

**DOI:** 10.3390/jcm15145426

**Published:** 2026-07-10

**Authors:** Omar Abureesh, Walid Sange, Qusai Al Zureikat, Toni Habib, Mohammad Abu-Shaban, Ghaith Rababah, Yousef Yousef, Ahmad Abdulraheem, Brendan Plann-Curley, Liliane Deeb

**Affiliations:** 1 Department of Internal Medicine, Staten Island University Hospital, Northwell Health, Staten Island, NY 10305, USA; 2 Department of Internal Medicine, MedStar Georgetown Washington Hospital Center, Washington, DC 20057, USA; 3 Department of Internal Medicine, Southeast Georgia Health System, Brunswick, GA 31520, USA; 4 Medical Library, Staten Island University Hospital, Northwell Health, Staten Island, NY 10305, USA; 5 Division of Gastroenterology and Hepatology, Department of Internal Medicine, Staten Island University Hospital, Northwell Health, Staten Island, NY 10305, USA

**Keywords:** GERD, lung cancer, lung adenocarcinoma, lung squamous cell carcinoma, small cell lung cancer, non-small cell lung cancer

## Abstract

**Background**: Gastroesophageal reflux disease (GERD) is a common problem that affects patients. The constant irritation of the esophageal mucosa, in addition to the potential aspiration, has been suggested as a predisposing factor for lung cancer (LC). This study aims to investigate the association between GERD and LC. **Methods**: An electronic search was conducted on EMBASE, CENTRAL, and PubMed databases from inception through November 2025. Studies evaluating the association between GERD and lung cancer in adult populations were included. Reviews, non-English articles, pediatric studies, and non-human studies were excluded. Twelve studies involving 2,900,965 patients met the inclusion criteria. Three types of common and random effect meta-analyses were utilized, namely meta-analysis of proportion, log(HR) and log(OR) models. Included studies were examined for risk of bias using the Newcastle–Ottawa scale. **Results**: Twelve articles describing 2,900,965 patients were found to be fit for inclusion. Male and female sexes were equally represented. On average, patients were 57.67 ± 10.02 years of age. Pooled proportion of co-occurring LC and GERD in patients was estimated at 64% [95% C.I.: 15–95%; *p* = 0.02], with significant heterogeneity (I^2^ = 96.8%, *p* < 0.01). Among studies calculating for hazard ratio (HR), an estimated pooled log(HR) of 1.01 [95% C.I.: 0.36–2.8; *p* = 0.98] with significant data heterogeneity (I^2^ = 88%, *p* < 0.01) was found. In comparison, five studies included estimates of odds ratio (OR), which resulted in a pooled log(OR) of 1.32 [95% C.I.: 1.2–1.46; *p* < 0.01], with less heterogeneity (I^2^ = 59.1%) which was marginally significant (*p* = 0.04). **Conclusions**: Our findings may suggest a possible association between LC and GERD; however, estimates were discordant across effect measures and heterogeneity was substantial, so this association should be interpreted with caution.

## 1. Introduction

According to estimates from the World Health Organization (WHO) in 2019, cancer is the first or second leading cause of death before the age of 70 years in 112 of 183 countries. Lung cancer (LC) ranks as the second most frequently diagnosed cancer and the primary cause of cancer-related deaths worldwide. According to GLOBOCAN 2020, an estimated 2.2 million new lung cancer cases (11.4%) and almost 1.8 million lung cancer deaths (18.0%) occurred in 2020 [1,2].

Cancer of the lung is divided into two main types: small cell lung cancer (SCLC) and non-small cell lung cancer (NSCLC). Several studies have shown that five-year survival rates for lung cancer at stages I and II range between 22–49% and 12–38%, respectively. Moreover, in advanced stage IV, they drop to 2–13%, affected by the frequent late diagnosis [3]. Approximately 30% of lung cancer cases are not attributable to known risk factors such as smoking, an unhealthy lifestyle, and rising air pollution [4]. It is crucial to identify potential modifiable risk factors to prevent its development. Among these factors, the recent literature has reported gastroesophageal reflux disease (GERD) as a possible contributor to the development of lung cancer [4,5,6]. GERD affects approximately 2.5% to 33.1% of the global population and is caused by chronic reflux of duodeno-gastric content into the esophagus, which results in epithelial damage, extending beyond the esophagus into airway epithelium [4].

This relationship has been attributed to various reasons explained by different pathophysiological hypotheses, including reflux aspiration and the common underlying risk factors depicting co-occurrence. The reflux aspiration hypothesis focuses on the fact that the acid refluxed into the esophagus causes chronic irritation and inflammation to esophageal and lung epitheliums, which results in activation of adaptive mechanisms such as metaplasia. Prolonged exposure of the airway epithelium to gastric acid and bile salts may lead to increased oxidative stress, activation of inflammatory mediators, and DNA damage that promote carcinogenesis that culminates in neoplasia and carcinoma [7]. On the other hand, some studies suggest that the use of proton-pump inhibitors, which is the main treatment for GERD, is associated with an increased risk of several cancers, including lung cancer [6]. These hypotheses and suggestions remain in need of deeper investigation and exploration. Moreover, there is a need to investigate the prevalence, underlying risk factors, and the bidirectional relationship between GERD and lung cancer. This study aims to investigate currently available evidence regarding the association between GERD and lung cancer.

## 2. Methods

### 2.1. Study Design

This research was conducted following the Preferred Reporting Items for Systematic Review and Meta-analysis (PRISMA) guidelines. The protocol with which the study was conducted was pre-registered at PROSPERO [CRD420251155756] (Supplementary File S1). The PRISMA checklist is included in Supplementary File S2.

### 2.2. Search Strategy

The search was conducted by a health sciences librarian, and results were uploaded to Covidence for review by authors. Comprehensive searches were performed on Embase, MEDLINE, PubMed, and CENTRAL, on 1 November 2025, from database inception onward. The following keywords were used to identify relevant articles: (“Lung cancer” OR “Lung Adenocarcinoma” OR “Adenocarcinoma of lung” OR “Non-esophageal cell lung cancer”) OR (“Non-Small cell lung cancer”) AND (“GERD” OR “GORD” OR “Gastroesophageal reflux diseases” OR “GER” OR “Aspiration” OR “acid reflux” OR “gastroesophageal reflux”). Additionally, Medical Subject Headings (Mesh) terms were used to identify all potentially relevant articles based on these indexed terms. To ensure a comprehensive search, we conducted a manual search following the screening of the articles to identify any potentially missing relevant articles through three approaches: (a) screening the reference list of the included articles, (b) screening “similar articles” to the included ones, through the “similar articles” options on PubMed, and (c) reviewing the included articles in the previously conducted meta-analysis.

### 2.3. Eligibility Criteria

Studies were included if they met the following criteria: [1] patients with any type of lung cancer, ref. [2] patients diagnosed with GERD, ref. [3] English papers. No restrictions were set on the date of publication. On the other hand, studies were excluded if they had one of the following criteria: [1] reviews, editorials, commentary [2], cancers other than lung cancer [3], patients not diagnosed with both conditions [1,2] simultaneously [4], pediatric patients (<18 years) [5], non-English papers [6], non-human models, and [7] unavailable full texts.

### 2.4. Study Selection

Following retrieval of the studies from the database search, citations were imported to EndNote for duplication removal. After duplicate removal, citations were exported into an Excel Sheet for screening. First, titles and abstracts of retrieved studies were screened against our prespecified eligibility criteria. Then, potentially relevant studies underwent full-text screening. This process was carried out by two independent reviewers [OA and WS] and any discrepancies were resolved through discussion with a third reviewer [TH]. The complete screening and filtration record is provided in the Supplementary File S3.

### 2.5. Data Extraction

Included papers were surveyed for the following: study characteristics (title, authors, country of investigation, year of publication, duration, etc.), patient characteristics (count, age, sex, race, etc.), proportion of patients with lung cancer and GERD, types of lung cancer reported. The primary outcome of interest was the association between lung cancer and GERD.

### 2.6. Quality Assessment

The methodological quality of the included studies was evaluated using the Newcastle–Ottawa scale for risk of bias assessment and a high risk of bias was defined as a score below 60%. This process was carried out by two reviewers [QZ and MA], and discrepancies were solved by group discussion.

### 2.7. Data Synthesis

The collected data were analyzed by quantitative and qualitative methods. Patient and study characteristics were reported narratively in tables. Proportions of GERD were analyzed using a fixed-effect model meta-analysis of proportion as the effect measure and 95% confidence intervals (CI). Log(OR) and log(HR) meta-analysis were utilized to estimate the pooled hazard ratio (HR) and odds ratio (OR). Because four included studies were genetic (GWAS/Mendelian randomization) analyses [8,9,10,11], all estimates were placed on a common log-odds scale before pooling. The three GWAS analyses drawing on the same consortium data (Dong, Yang and Wu; each *n* = 602,604) [9,10,11] were combined into a single estimate to avoid double-counting. A pre-specified sensitivity analysis pooled observational and genetic estimates separately. When significant heterogeneity was detected (I^2^ > 50%), we adopted a random-effects model. Statistical significance was set at *p* < 0.05. Heterogeneity was assessed using I^2^ and Cochran’s Q statistic, interpreted according to the Cochrane Handbook for Systematic Reviews. We identified studies causing heterogeneity using the leave-one-out method. If significant heterogeneity persisted after switching to a random-effects model, the relevant study was excluded from the synthesis. Statistical analyses were conducted using R software (version 4.12 of the meta package; R Foundation for Statistical Computing; Vienna, Austria). Analysis of publication bias using funnel plot asymmetry was not applicable with meta-analysis of proportions, nor with log(HR) and log(OR), since the number of included studies did not amount to 10.

## 3. Results

**Figure 1:**
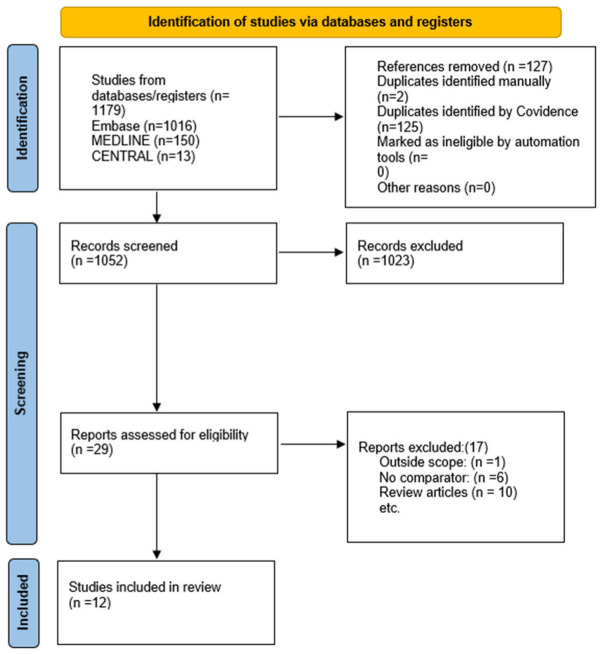
A PRISMA chart illustrates the filtration process of the search results.

Our search yielded 1179 articles which were filtered through titles and abstracts into 29 articles, which were then subjected to full-text screening, yielding 12 articles that met our inclusion criteria in the present study. Figure 1 shows a PRISMA diagram illustrating the filtration process.

**Figure 2:**
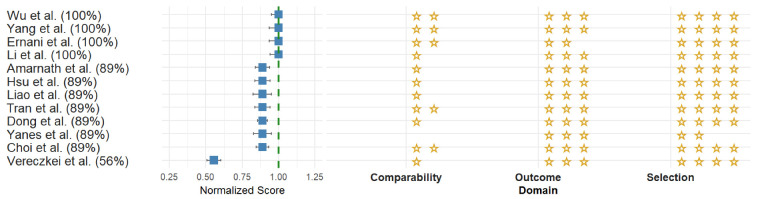
Newcastle–Ottawa scale risk of bias assessment of included studies [4,8,9,10,11,12,13,14,15,16,17,18].

The full-text articles that were excluded are the 17 articles in Figure 1. The included articles describe 2,900,965 patients across the world. Studies were generally designed as cohort studies; however, four studies were conducted as Genome-Wide Association Studies (GWAS). Of the included articles, three studies were based on the same population, namely those of Dong et al., Yang et al. and Wu et al. [9,10,11] These studies investigated three different outcomes in the setting of GERD. Yang et al. had the narrowest scope, focusing on the association between GERD and lung cancer. Meanwhile, Dong et al. focused on the association between GERD and respiratory diseases including lung cancer, and Wu et al. investigated GERD and its relationship to cancers in general, including lung cancers. For this purpose, although these studies were included, only Yang et al.’s findings were utilized for analysis, as they have the highest-precision inclusion criteria in the context of this article. Table 1 summarizes the characteristics of the included studies. The included studies were at low risk of bias according to the Newcastle–Ottawa scale, except for Vereczkei et al.’s, which had a high risk of bias [12]. Figure 2 illustrates the risk of bias assessment results of the included studies.

Ernani et al.’s study evaluated survival characteristics among patients with established SCLC rather than incident lung cancer risk, limiting comparability with other included studies. Included patients were of varying races. Both genders were equally represented (male: 50.3%, female: 49.7%). Patients included had an average age of 57.67 ±10.02 years, and information on these patients was collected over an average period of 15.19 ± 7.7 years. Table 2 highlights the main characteristics of the included patients.

Included studies differed in their targeted study population. Some targeted GERD prevalences in patients with established LC patients, while some targeted GERD patients and the prevalence of LC amongst them. One study (Liao et al.) targeted the general population, collecting information about the co-occurrence of GERD and LC compared to LC-free or GERD-free individuals. In Table 3, population features in the investigated studies are presented.

**Figure 3:**
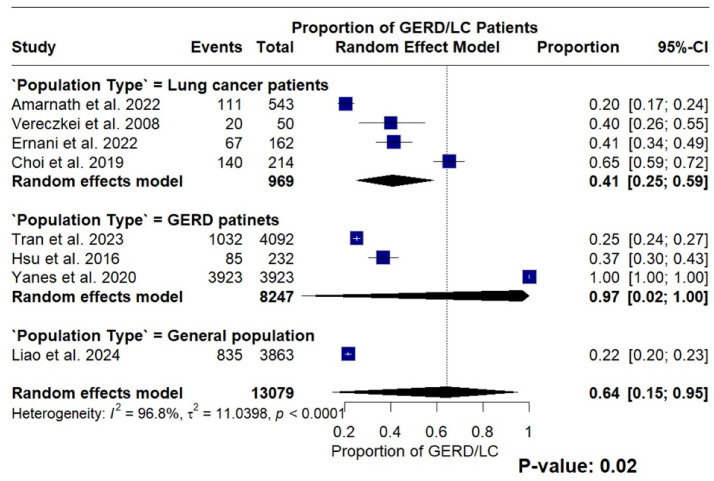
Meta-analysis of proportion of GERD incidence in LC patients [4,13,14,16,17,18].

After alignment, meta-analysis of the proportion of LC patients with co-occurring GERD was applicable, excluding studies where LC prevalence in GERD patients was investigated, namely by Lui et al. and Dong et al. The pooled proportion of LC patients with co-occurring GERD was found at 64% [95% C.I.: 15–95%; *p* = 0.02]. Data was significantly heterogenous (I^2^ = 96.8%, *p* < 0.01), and subgrouping according to the targeted population showed all subgroups suffering from heterogeneity. Figure 3 illustrates the forest plot of meta-analysis of GERD incidence in LC patients.

**Figure 4:**
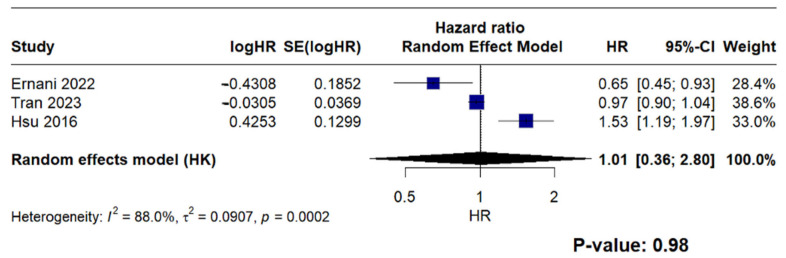
Forest plot showing meta-analysis of log(HR) of co-occurrence of GERD in LC patients [15,16,17].

**Figure 5:**
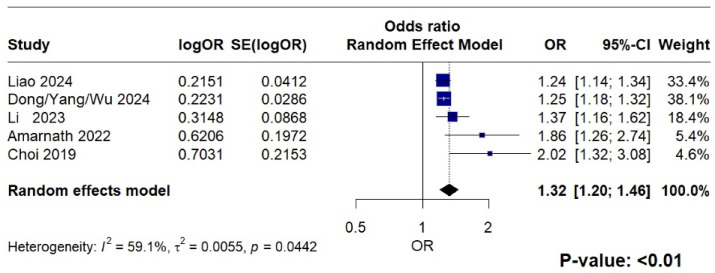
Forest plot showing meta-analysis of log(OR) of co-occurrence of GERD in LC patients [4,8,9,10,11,13,18].

Most included studies reported the relationship between GERD and LC and an OR of GERD in LC patients. Risk estimates were utilized to represent the potential prevalence of GERD in patients with various types of LC. To calculate risk estimates for pooled evidence, two random-effects model meta-analyses were conducted, one for hazard ratio (HR) and one for odds ratio (OR). As shown in Figure 4, three studies reporting HR were analyzed, resulting in an estimated pooled log(HR) of 1.01 [95% C.I.: 0.36–2.8; *p* = 0.98] with significant data heterogeneity (I^2^ = 88%, *p* < 0.01). Regarding log(HR), results were insignificant and non-deterministic. On the other hand, Figure 5 illustrates meta-analysis of included articles reporting OR of GERD development in LC patients. Five studies included estimates of OR, and calculated pooled log(OR) was 1.32 [95% C.I.: 1.2–1.46; *p* < 0.01]. Although heterogeneous, in analyzing for log(OR), data showed less heterogeneity (I^2^ = 59.1%) which was statistically marginally significant (*p* = 0.04). Thus, significant positive logarithmic odds of cooccurrence of LC in GERD patients were observed. In sensitivity analysis, the observational-only pooled OR was 1.60 (95% CI 1.12–2.28; I^2^ = 76.9%; *p* = 0.010) and the genetic/MR-only pooled OR was 1.26 (95% CI 1.20–1.33; I^2^ = 0.7%; *p* < 0.001). Both were significant and directionally consistent with the primary estimate (OR 1.32), and the genetic subset was homogeneous, indicating the pooled result is not an artefact of combining study designs.

## 4. Discussion

The recent literature has pointed out the potential association between GERD and LC. However, pooled evidence and quantitative analysis on the direction and size of this relationship remain lacking. Unlike prior meta-analyses, the present review additionally incorporated Mendelian randomization and GWAS-based evidence, enabling broader exploration of potential biologic associations and confounding structures. The present study summarizes evidence on 2,900,965 patients across the world, which is a significant amount of study subjects. Most studies focused on Western populations, i.e., the United States, Europe, which is understandable as the availability of nationwide registrars is limited elsewhere.

On average, patients were 57.67 ± 10.02 years old, which corresponds to a combination of the average age of lung cancer at 70 years old and the average age of GERD diagnosis at 40–50 years old [19,20]. Pooled proportion of GERD co-occurring LC patients was found to be 64% [95% C.I.: 15–95%; *p* = 0.02; I^2^ = 96.8%; *p* < 0.01]. The focus on GERD predisposing LC highlights the fact that a large proportion of LC patients are already GERD patients. A study on GERD diagnosis in LC patients reported a 23% occurrence of GERD and LC [17], but the small size of said study limits the validity of their findings. This, however, can be affected by multiple confounding variables; for instance, smoking is a strong factor resulting in both GERD and LC development. Although attempted, smoking status was not provided in most included studies. Therefore, a subgroup analysis on smoker vs. nonsmoker patients was inapplicable. Yang et al. focused on this issue, suggesting a strong confounding effect of smoking and alcohol on the emergence of GERD and LC, starting with GERD due to epithelial irritation, and developing into LC after sequences of metaplasia [10]. Smoking is, however, only one of several shared confounders. Alcohol use, obesity and elevated body mass index, chronic obstructive pulmonary disease and other chronic airway inflammation, Helicobacter pylori infection, socioeconomic status, age, and proton-pump inhibitor exposure are each independently associated with GERD, with lung cancer, or with both, and none could be consistently accounted for across the included studies.

Three studies reported HR of LC coinciding with GERD, and an estimated pooled HR of 1.01 [95% C.I.: 0.36–2.8; *p* = 0.98] with significant data heterogeneity (I^2^ = 88%, *p* < 0.01) was found. This result was statistically insignificant, and judging by the study design of included studies, since most studies included patients with GERD who developed lung cancer later, this estimation can be rather inaccurate. Five studies included estimates of OR, and calculated pooled log(OR) was 1.32 [95% C.I.: 1.2–1.46; *p* < 0.01; I^2^ = 59.1%, *p* = 0.04). The significantly positive log(OR) of 1.32 suggests a strong association between LC and GERD, the direction of which would be easy to describe usually (GERD corresponds to higher incidence of LC). While pooled OR estimates suggested a positive association, HR-based analyses did not demonstrate statistically significant results, highlighting inconsistency across study designs and effect measures and because of methodological diversity among included studies, pooled estimates should be interpreted as exploratory. Despite describing starting with GERD, and evaluating its presence in LC patients, most studies included in this review included populations with prior diagnosis of LC, which would make the direction of this relationship difficult to determine. Within these limits, the pooled odds analysis is consistent with an association between the two conditions, although its strength and direction cannot be firmly established.

Irritation to gastric and esophageal mucosa has been described as a risk factor for lung cancer in multiple studies. Aspiration, Helicobacter pylori infection, and GERD were all found to be correlating factors [21,22,23].

These mechanisms offer a biologically plausible basis for the association seen in our pooled OR analysis, but they do not establish that GERD causes lung cancer, and our data do not support such a claim. Two features of the present evidence limit any causal interpretation. First, the pooled HR was not statistically significant and was highly heterogeneous, so the magnitude and even the direction of any prospective risk remain uncertain. Second, most included studies were based on medical records in which GERD and lung cancer were ascertained together, so the temporal sequence between the two could not be reliably established despite assertions that GERD preceded lung cancer. The discrepancy between the significant OR and the non-significant HR is therefore more likely to reflect differences in study design, effect measure, and residual confounding than a true biological gradient. For these reasons, any suggestion that treating GERD or limiting epithelial irritation could reduce lung cancer risk should be regarded as hypothesis-generating only and would require confirmation in studies designed to capture exposure before outcome.

The findings of the present study remain limited. Due to the large number of cohorts investigated, clear diagnostic criteria were generally not provided. Moreover, most studies included patients by accessing their medical records and identifying their GERD and LC diagnosis, which would compromise the ability to identify which disease came about first, although they claimed to have diagnosed GERD prior to LC. Another issue is the measure of effect included in the meta-analysis, as meta-analysis of log(OR) and log(HR) carries statistical assumptions that are usually complex and not easily satisfied. These limitations are closely tied to the pathophysiology that motivates the GERD lung cancer hypothesis. The proposed mechanisms—chronic micro-aspiration of gastric contents, sustained airway inflammation, oxidative DNA damage, and metaplastic change—are slow processes that would act over many years, and they share upstream drivers such as smoking, obesity, and chronic obstructive pulmonary disease that independently raise the risk of both conditions. A meta-analysis can only be as informative as the primary studies it pools, and the available studies were not designed to separate these intertwined pathways. Because most relied on record-based diagnoses captured at a single point, exposure and outcome were effectively measured together, which makes the latency required by the biological model impossible to observe and leaves pooled estimates vulnerable to reverse causation and immortal time bias. The marked statistical heterogeneity we observed (I^2^ up to 96.8%) is therefore likely to be clinical and methodological rather than purely statistical in origin, and pooling across such diverse designs can obscure as much as it reveals. Future studies could reduce this heterogeneity in several concrete ways: by adopting a uniform, guideline-based definition of GERD (for example, endoscopically or pH-confirmed disease rather than diagnostic codes alone); by using prospective cohort designs with a clear temporal window in which GERD is documented before any lung cancer diagnosis; by reporting results separately for histological subtypes of lung cancer; by pre-specifying adjustment for shared confounders, particularly smoking pack-years, alcohol use, body mass index, and chronic obstructive pulmonary disease; and by harmonizing the effect measure reported. Prospective registration and standardized reporting in line with current systematic review guidance would further allow such studies to be combined more meaningfully. Until evidence of this kind is available, the relationship between GERD and lung cancer should be regarded as an association in need of confirmation rather than an established causal pathway.

## 5. Conclusions

This systematic review and meta-analysis of 2,900,965 patients suggest a possible association between gastroesophageal reflux disease and lung cancer, supported by a significant pooled odds ratio (1.32) but tempered by a null pooled hazard ratio (1.01) and substantial heterogeneity. These findings are hypothesis-generating rather than confirmatory: the data do not establish that GERD causes or increases the incidence of lung cancer, because most included studies could not confirm that GERD preceded lung cancer and could not fully account for shared confounders, most importantly smoking. Confirming a causal relationship will require prospective cohorts that apply a uniform, objective definition of GERD (for example, endoscopically or pH-confirmed disease rather than diagnostic codes alone); document GERD before any lung cancer diagnosis; adjust for shared confounders, including quantitative smoking exposure (pack-years), alcohol, body mass index and COPD; analyze histological subtypes separately; and harmonize the reported effect measure so that estimates can be pooled consistently.

## Figures and Tables

**Table 1:** Characteristics of included articles.

Article ID	Year of Publication	Country	Sample Size	Study Design *	Study Setting *	Population Type
Choi et al. [13]	2019	Republic of Korea	1070	Cohort	General population	Lung cancer
Vereczkei et al. [12]	2008	Hungary	50	Cohort	Hospital-based	Lung cancer
Yanes et al. [14]	2020	Nordic countries	812,617	Cohort	EMR	GERD
Ernani et al. [15]	2022	USA	162	Cohort	EMR	Lung cancer
Tran et al. [16]	2023	South Korea	41,044	Cohort	EMR	GERD
Liao et al. [4]	2024	UK	458,691	Cross-sectional	EMR	General
Hsu et al. [17]	2016	Taiwan	76,369	Cohort	EMR	GERD
Amarnath et al. [18]	2022	USA	1083	Case–control	Hospital-based	Lung cancer
Li et al. [8]	2023	China	367,441	GWAS	General population	Lung cancer
Dong et al. [9]	2024	China	602,604	GWAS	EMR	GERD
Yang et al. [10]	2023	China		GWAS	EMR	GERD
Wu et al. [11]	2024	UK		GWAS	EMR	GERD
Total			2,900,965			

* EMR: Electronic medical record; GWAS: Genome-Wide Association Study.

**Table 2:** Characteristics of included patients.

Article ID	Sample Size	Male/Female Ratio	Ethnicity	Age (Mean ± Std)	Duration (Years)
Choi et al. [13]	1070	44.4%/55.6%	Korean	N/A	13
Vereczkei et al. [12]	50	32%/18%	European	60.2 ± 6.8	N/A
Yanes et al. [14]	812,617	52.45%/47.55%	European	56.7 ± 11.1	34
Ernani et al. [15]	162	42%/58%	Caucasian	63.7 ± 10.4	19
Tran et al. [16]	41,044	50.1%/49.9%	Korean	54.4 ± 8.8	13
Liao et al. [4]	458,691	46.8%/53.2%	European	60 ± 8.15	11.54
Hsu et al. [17]	76,369	49%/51%	N/A	55.9 ± 3.87	13
Amarnath et al. [18]	1083	26%/74%	N/A	72.9 ± 13.1	8
Li et al. [8]	367,441	N/A	European	N/A	N/A
Dong et al. [9]	602,604	N/A	European	N/A	10
Yang et al. [10]					
Wu et al. [11]					
Total	2,900,965	50.3%/49.7%		57.67 ± 10.02	15.19 ± 7.7

**Table 3:** Population features of included studies.

Study ID	Sample Size	No. of GERD Patients	No. of Lung Cancer Patients	Subtype of Lung Cancer *				Population Type
				LUAD	LUSC	SCLC	Other/NSCLC	
Choi et al. [13]	1070	140	214	-	-	-	214	Lung cancer
Vereczkei et al. [12]	50	20	50	25		25	-	Lung cancer
Yanes et al. [14]	812,617	3923	3923	1916	1240	767	-	GERD
Ernani et al. [15]	162	67	162	-	-	162	-	Lung cancer
Tran et al. [16]	41,044	1032	4092	-	-	-	4092	GERD
Liao et al. [4]	458,691	835	3863	360	757	1560	1186	General
Hsu et al. [17]	76,369	85	232	-	-	-	232	GERD
Amarnath et al. [18]	1083	111	543	505	33	5	-	Lung cancer
Li et al. [8]	907,275	78,707	30,756	3442	3275	461	23,578	Lung cancer
Dong et al. [9]	602,604	129,080	2671	11,273	7426	2664	10,571	GERD
Total	2,900,965 (100%)	214,000 (7.37%)	46,506 (1.6%)	17,521 (0.6%)	12,731 (0.44%)	5644 (0.19%)	39,873 (1.37%)	

* LUAD: Lung Adenocarcinoma; LUSC: Lung Squamous Cell Carcinoma; SCLC: Small Cell Lung Cancer; NSCLC: Non-Small Cell Lung Cancer.

## Data Availability

Data is available upon reasonable request from the corresponding author.

## Supplementary Materials

The following supporting information can be downloaded at https://www.mdpi.com/article/10.3390/jcm15145426/s1, File S1: PROSPERO protocol [24]; File S2: PRISMA checklist [25] and File S3: the full filtration sheet.
