# Supplementary material for: The Association Between Gastroesophageal Reflux Disease and Lung Cancer: A Systematic Review and Meta Analysis
Source: J Clin Med. 2026 Jul 10;15(14):5426. doi: 10.3390/jcm15145426 (PMC13412617; doi:10.3390/jcm15145426)
Supplement: Supplementary file 1 [file jcm-15-05426-s001.zip › File S1 PROSPERO.pdf]

NIHR | National Institute for Health and Care Research

PROSPERO
International prospective register of systematic reviews

The Association Between Gastroesophageal Reflux Disease and Lung Cancer: A Systematic review and Meta analysis

Omar Abureesh, Liliane Deeb

Citation
Omar Abureesh, Liliane Deeb. The Association Between Gastroesophageal Reflux Disease and Lung Cancer: A Systematic review and Meta analysis . PROSPERO 2025 CRD420251155756. Available from https://www.crd.york.ac.uk/PROSPERO/view/CRD420251155756.

REVIEW TITLE AND BASIC DETAILS

Review title

The Association Between Gastroesophageal Reflux Disease and Lung Cancer: A Systematic review and Meta analysis

Condition or domain being studied

Gastro-esophageal reflux disease (GERD) is a condition that develops when the reflux of stomach contents causes troublesome symptoms and complications. GERD is a global issue that affects both children and adults. GERD is associated with the risk of many lung diseases, such as cancer, obstructive and restrictive lung diseases. However, the association between the lung cancer and GERD is still under investigated and need more studies and exploration.

Rationale for the review

Gastro-esophageal reflux disease (GERD) is a condition that develops when the reflux of stomach contents causes troublesome symptoms and complications. GERD is a global issue that affects both children and adults. GERD is associated with the risk of many lung diseases, such as cancer, obstructive and restrictive lung diseases. However, the association between the lung cancer and GERD is still under investigated and need more studies and exploration.

This review aims to investigate the relationship between lung cancer and GERD and explore the common and predisposing risk factors for both.

Review objectives

To analyze the present evidence on the association of GERD and lung cancer , while taking into account the demographic characteristics of affected individuals, their clinical characteristics, diagnostic tools, and underlying risk factors.

Keywords
Lung cancer; GERD; GORD; Reflux; Gastroesophageal reflux disease

Country
United States of America

ELIGIBILITY CRITERIA

Population

Included
Patient with any type of lung cancer

Excluded
pediatric age

cancer other than lung cancer

Intervention(s) or exposure(s)

Included
GERD

GORD

acid reflux

Excluded
2. Reported presence of Gastroesophageal Reflux Disease (GERD) diagnosed by one of the following: Clinical diagnosis by a physician , Validated questionnaire administrative codes, Pharmacological proxy: Regular use of acid-suppressive medications or Self-report of a prior GERD diagnosis in large cohort studies and Incidence of lung cancer among GERD patients

Comparator(s) or control(s)

Included
PICO tags selected: Normal diet

Study design
Only nonrandomized study types will be included.

Included
1. Observational studies including longitudinal Prospective or retrospective cohorts studies, cross sectional studies and population-based and hospital-based case-Control Studies

Excluded
1. Reviews, editorials, commentaries, case reports, case series (with fewer than 10 subjects), and animal studies.

Context
studies on GERD and lung cancer in the same patients , all over the world, including all types of lung cancer

TIMELINE OF THE REVIEW

Date of first submission to PROSPERO
30 September 2025

Review timeline
Start date: 26 May 2025. End date: 1 November 2025.

Date of registration in PROSPERO
05 October 2025

AVAILABILITY OF FULL PROTOCOL

Availability of full protocol
A full protocol has been written and uploaded to PROSPERO. The protocol will be made available after the review is completed.

SEARCHING AND SCREENING

Search for unpublished studies
Only published studies will be sought.

Main bibliographic databases that will be searched
The main databases to be searched are *Embase - Embase via Ovid*, *MEDLINE*, *PubMed* and *Scopus*.

Search language restrictions
The review will only include studies published in English.

Search date restrictions
There are no search date restrictions.

Other methods of identifying studies
Other studies will be identified by: *contacting authors or experts*, *looking through all the articles that cite the papers included in the review ("snowballing" or forward citation searching)* and *reference list checking (backward citation searching)*.

Link to search strategy
A full search strategy is available in the full protocol as described in the *Availability of full protocol* section

Selection process
Studies will be screened independently by at least two people (or person/machine combination) with a process to resolve differences.

DATA COLLECTION PROCESS

Data extraction from published articles and reports
Data will be extracted independently by at least two people (or person/machine combination) with a process to resolve differences.

Authors will be asked to provide any required data not available in published reports.

Study risk of bias or quality assessment
Risk of bias will be assessed using: *Newcastle-Ottawa*

Data will be assessed independently by at least two people (or person/machine combination) with a process to resolve differences.

Additional information will be sought from study investigators if required information is unclear or unavailable in the study publications/reports.

Reporting bias assessment
Risk of bias due to missing results will be assessed

Certainty assessment
we will consider any statistical test results >0.05 as statical insignificant

heterogeneity among the study will assessed by p value too

publication bias will be assessed by funnel plot

OUTCOMES TO BE ANALYSED

Main outcomes
To analyze the present evidence on the association of GERD and lung cancer , while taking into account the demographic characteristics of affected individuals, their clinical characteristics, diagnostic tools, and underlying risk factors. Risk ratio, relative risk and Odd ratio will be used

Additional outcomes
There are no additional outcomes.

PLANNED DATA SYNTHESIS

Strategy for data synthesis
at beginning we will describe the patients characteristic narratively and by simple descriptive statistics like mean age of the all patient, male to female ratio, percentage of each lung cancer

after that the association between lung cancer and GERD will be pooled to investigate if there is any relationship existed.

then, the risk factor for both of lung cancer and GERD will be investigated to explore the common risk factors

then if applicable, the severity of lung cancer will be correlated to the severity, duration, complications of the GERD

finally, we will investigate the confounding factors like treatment used in GERD and its association with lung cancer

CURRENT REVIEW STAGE

Stage of the review at this submission

Review stage	Started	Completed
Pilot work	✓	
Formal searching/study identification	✓	
Screening search results against inclusion criteria		
Data extraction or receipt of IPD		
Risk of bias/quality assessment		
Data synthesis		

Review status
The review is currently planned or ongoing.

Publication of review results
Results of the review will be published.

REVIEW AFFILIATION, FUNDING AND PEER REVIEW

Review team members
Dr Omar Abureesh (review guarantor and contact) ORCID: 0000-0002-3158-4120. Northwell Health. United States of America.

No conflict of interest declared.

Professor Liliane Deeb. Staten Island University Hospital. United States of America.

No conflict of interest declared.

Named contact
Dr Omar Abureesh (oabureesh@northwell.edu). ORCID: 0000-0002-3158-4120. Northwell Health. United States of America.

Review affiliation
Staten Island University Hospital Northwell Health

Funding source
Review has no funding and no agreed support from an academic institution and is done in authors' own time.

Peer review
There has been no peer review of this planned review.

ADDITIONAL INFORMATION

Review conflict of interest
Declared individual interests are recorded under team member details.. No additional interests are recorded for this review.

Medical Subject Headings
Gastroesophageal Reflux; Humans; Lung Neoplasms; Risk Factors

SIMILAR REVIEWS

Check for similar records already in PROSPERO

PROSPERO identified a number of existing PROSPERO records that were similar to this one (last check made on 29 September 2025). These are shown below along with the reasons given by that the review team for the reviews being different and/or proceeding.

- Association between gastroesophageal reflux disease and atrophic gastritis: A Systematic Review and Meta-analysis [published 17 September 2022] [CRD42022358436]. The review was judged **not to be similar**
- Association between gastroesophageal reflux and upper aero-digestive tract (UADT) cancer risk: a systemic review and meta-analysis [published 7 November 2024] [CRD42024602545]. The review was judged **not to be similar**
- The association between salivary pepsin and gastroesophageal reflux disease: A meta-analysis. [published 29 February 2024] [CRD42024513901]. The review was judged **not to be similar**

PROSPERO version history

- Version 1.0, published 05 Oct 2025

Disclaimer

The content of this record displays the information provided by the review team. PROSPERO does not peer review registration records or endorse their content.

PROSPERO accepts and posts the information provided in good faith; responsibility for record content rests with the review team. The guarantor for this record has affirmed that the information provided is truthful and that they understand that deliberate provision of inaccurate information may be construed as scientific misconduct.

PROSPERO does not accept any liability for the content provided in this record or for its use. Readers use the information provided in this record at their own risk.

Any enquiries about the record should be referred to the named review contact
